# Supplementary material for: Worldwide Selection Footprints for Drought and Heat in Bread Wheat (Triticum aestivum L.)
Source: Plants (Basel). 2022 Sep 1;11(17):2289. doi: 10.3390/plants11172289 (PMC9460392; doi:10.3390/plants11172289)
Supplement: Supplementary file 1 [file plants-11-02289-s001.zip › plants-1871600-supplementary.pdf]

Supplementary Information

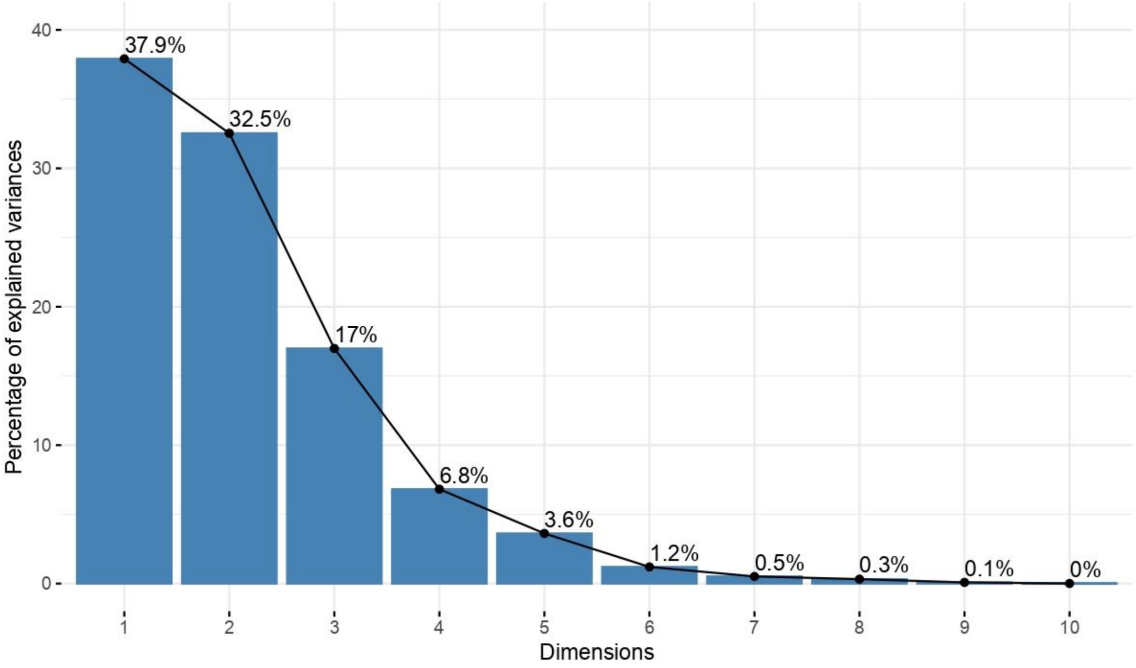

a)

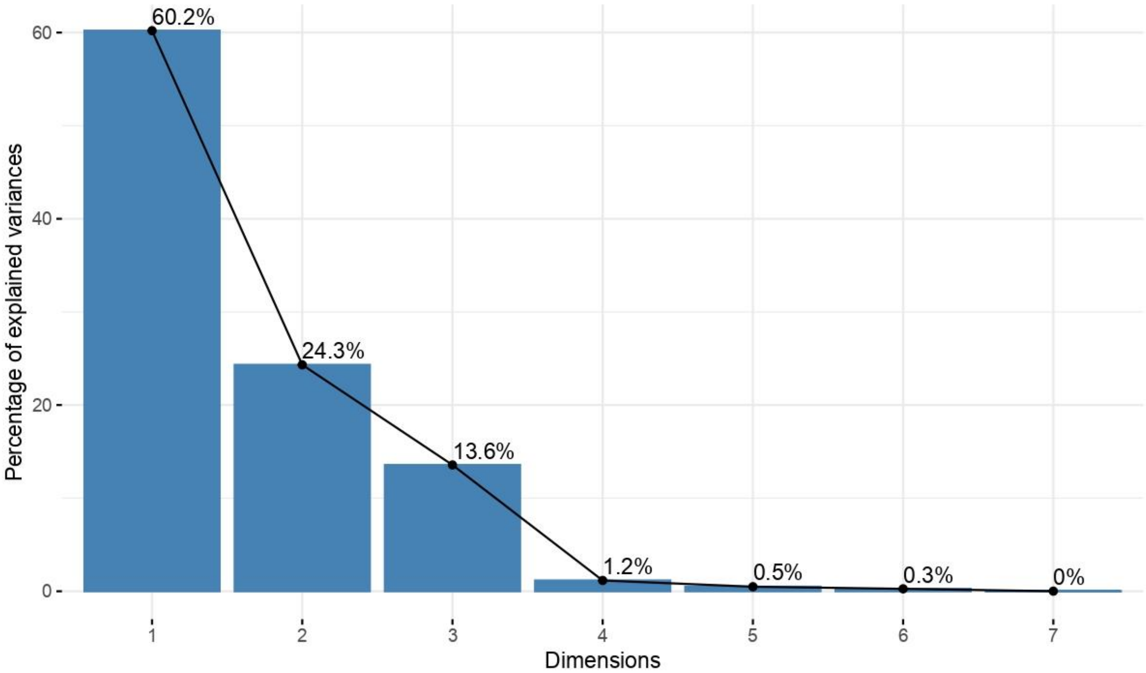

b)

Figure S1. Contributions to the total variation of each component for the evaluated variables in the PCA. a) Bioclimatic variables related to aridity; b) Temperature related variables, calculated indices, AP, and ELEV.

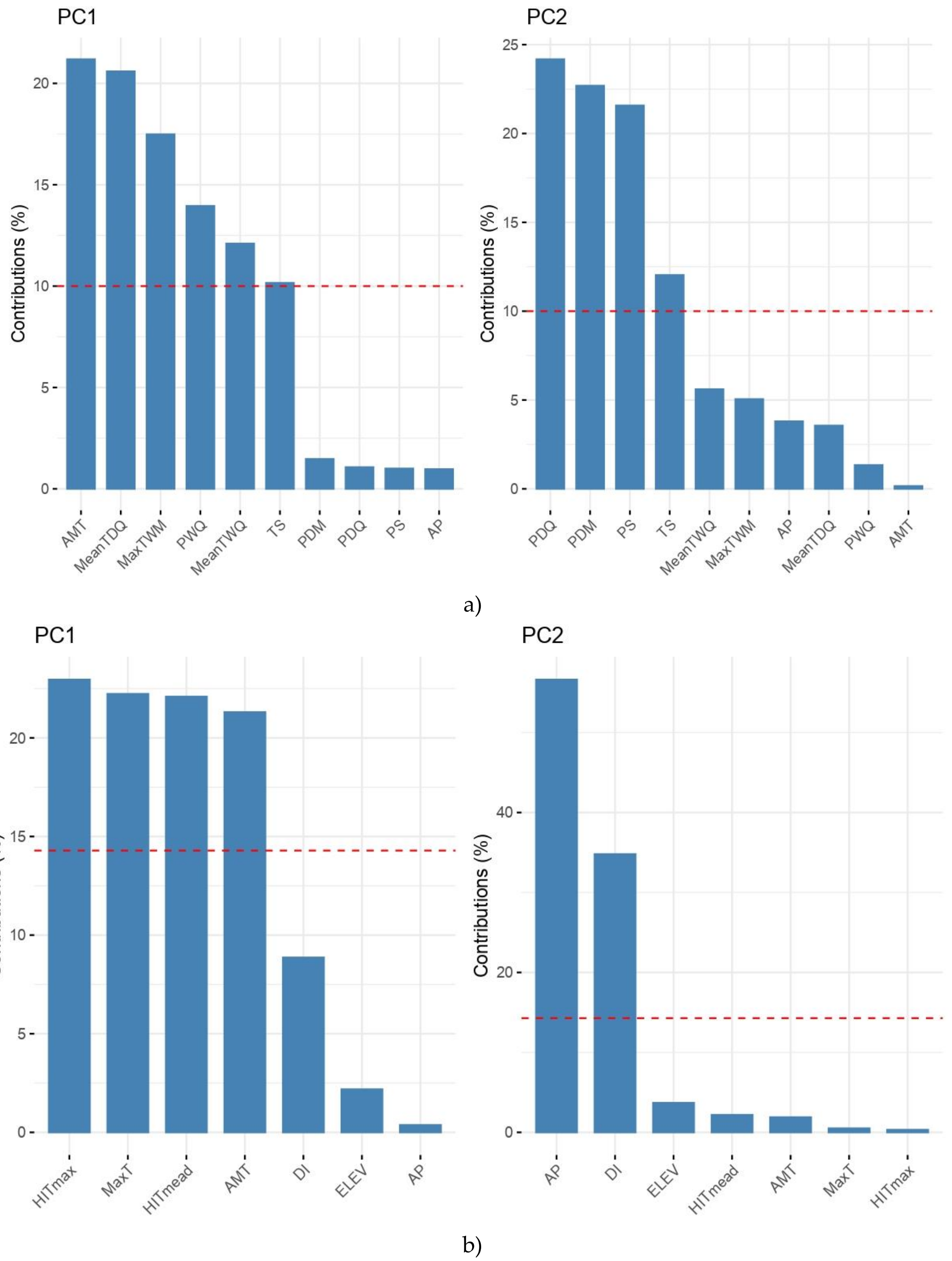

Figure S2. Contributions to component principals 1 and 2 for the evaluated variables in the PCA. a) Bioclimatic variables related to aridity; b) Temperature related variables, calculated indices, AP, and ELEV.

Table S1. Detection of associated SNPs for seven climatic variables.

Var	Chr	No.	SNP	Physical position (bp)	*P-value*	SNPs	Genes
AMT	1A	1	108825112|F|0-19:T>A-19:T>A	277825486	6.60E-09	2	1
		2	108891114|F|0-33:A>G-33:A>G	388440316	5.89E-09		1
	1D	3	108739422|F|0-31:T>A-31:T>A	2113561	1.49E-06	1	1
	2D	4	108968402|F|0-23:C>T-23:C>T	79989524	9.63E-07	1	2
	3D	5	107698139|F|0-48:C>T-48:C>T	97153088	4.42E-07	2	1
		6	108308207|F|0-18:G>A-18:G>A	546672913	2.49E-07		2
	4D	7	109431634|F|0-54:G>A-54:G>A	370691683	5.90E-10	1	3
	5D	8	108305241|F|0-68:A>G-68:A>G	379130055	1.29E-06	1	1
						Total	**12**
MaxT	1A	1	108825112|F|0-19:T>A-19:T>A	277825486	8.02E-08	2	1
		2	108891114|F|0-33:A>G-33:A>G	388440316	1.32E-16		1
	3A	3	106510612|F|0-30:C>T-30:C>T	502971897	2.05E-07	3	2
		4	108476623|F|0-32:T>A-32:T>A	507114154	4.55E-07		2
		5	108028914|F|0-20:A>G-20:A>G	574516154	1.86E-07		2
	4D	6	109431634|F|0-54:G>A-54:G>A	370691683	1.09E-16	1	3
	5D	7	108652995|F|0-58:G>C-58:G>C	528660566	1.50E-08	1	1
	6A	8	109126792|F|0-49:C>T-49:C>T	5854616	1.23E-06	1	2
	6D	9	108582786|F|0-49:T>G-49:T>G	143959936	1.95E-12	1	2
	7A	10	108830300|F|0-9:T>G-9:T>G	64789408	3.05E-10	2	2
		11	109126469|F|0-35:G>A-35:G>A	662017143	8.41E-07		2
	7B	12	107878167|F|0-16:G>A-16:G>A	107522176	1.30E-06	1	2
						Total	**22**
AP	1A	1	108256081|F|0-24:C>G-24:C>G	588647447	1.25E-07	1	1
	2A	2	109058266|F|0-7:C>T-7:C>T	640221452	1.32E-07	1	2
	2B	3	107797386|F|0-11:T>G-11:T>G	91596926	1.14E-09	2	1
		4	107593284|F|0-50:T>A-50:T>A	768567114	3.32E-07		1
	4A	5	108145069|F|0-52:C>A-52:C>A	714179675	1.02E-06	1	2
	5B	6	109305833|F|0-34:C>G-34:C>G	526249013	1.56E-06	1	1
	6B	7	109177937|F|0-11:A>G-11:A>G	223441723	1.81E-07	2	1
		8	109523315|F|0-24:C>T-24:C>T	269818400	1.26E-06		0
						Total	**9**
PS	1D	1	107874524|F|0-37:G>C-37:G>C	485539732	1.89E-07	1	3
	2A	2	108020469|F|0-43:T>C-43:T>C	711971015	1.78E-08	2	2
		3	108514047|F|0-10:A>C-10:A>C	729345577	3.13E-08		2
	2B	4	107794074|F|0-23:T>C-23:T>C	76022529	9.09E-08	2	2
		5	109021888|F|0-34:T>C-34:T>C	795754781	1.02E-07		1
	3B	6	108953425|F|0-21:A>G-21:A>G	50526285	1.78E-06	3	1
		7	107601308|F|0-23:G>A-23:G>A	741467423	1.38E-09		0
		8	108516380|F|0-24:T>C-24:T>C	758205945	5.53E-10		1

Table S1. Continued.

Var	Chr	No.	SNP	Physical position (bp)	*P-value*	SNPs	Genes
PS	4B	9	106772473|F|0-11:T>G-11:T>G	39759168	4.41E-07	1	1
	5A	10	107001591|F|0-37:T>C-37:T>C	619468262	3.81E-07	1	2
	5B	11	108348543|F|0-58:A>G-58:A>G	548120559	2.48E-08	2	1
		12	109119258|F|0-68:T>C-68:T>C	577227418	4.69E-09		2
	6B	13	109354013|F|0-58:A>G-58:A>G	485290761	2.50E-08	1	2
	7A	14	108619258|F|0-17:C>T-17:C>T	552606647	1.35E-08	1	1
	7B	15	108981313|F|0-8:C>T-8:C>T	650581291	5.17E-08	1	1
						Total	**22**
HITmead	1A	1	108891114|F|0-33:A>G-33:A>G	388440316	9.11E-18	1	1
	1B	2	108853213|F|0-26:C>T-26:C>T	201700273	4.52E-07	1	1
	1D	3	107880401|F|0-66:C>T-66:C>T	413939387	1.53E-07	1	2
	2B	4	107488994|F|0-17:T>C-17:T>C	29129965	1.44E-08	1	3
	3B	5	108146856|F|0-65:T>A-65:T>A	764282419	2.77E-07	1	1
	3D	6	107698139|F|0-48:C>T-48:C>T	97153088	1.25E-08	1	1
	4A	7	109242168|F|0-13:A>G-13:A>G	598521397	4.39E-08	1	2
	4B	8	108145974|F|0-24:T>C-24:T>C	56276689	3.44E-07	1	1
	4D	9	109363420|F|0-34:G>A-34:G>A	62303691	1.34E-08	3	2
		10	108773963|F|0-37:C>T-37:C>T	97959582	1.59E-06		2
		11	109431634|F|0-54:G>A-54:G>A	370691683	1.50E-08		3
	5B	12	109240982|F|0-38:G>C-38:G>C	68925700	2.02E-12	2	1
		13	108773279|F|0-51:C>T-51:C>T	530916378	8.04E-07		2
	7A	14	107178221|F|0-24:T>G-24:T>G	628906923	1.54E-06	1	2
						Total	**24**
HITmax	1A	1	108825112|F|0-19:T>A-19:T>A	277825486	8.48E-09	2	1
		2	108891114|F|0-33:A>G-33:A>G	388440316	1.94E-11		1
	2B	3	108024073|F|0-47:C>T-47:C>T	38321752	3.81E-09	1	2
	3A	4	108476623|F|0-32:T>A-32:T>A	507114154	1.68E-08	2	2
		5	108028914|F|0-20:A>G-20:A>G	574516154	7.31E-11		2
	3B	6	109122135|F|0-40:A>G-40:A>G	535641207	9.91E-07	1	1
	3D	7	108308207|F|0-18:G>A-18:G>A	546672913	4.37E-07	2	2
		8	109610030|F|0-20:C>T-20:C>T	575639014	6.69E-08		2
	4A	9	109242168|F|0-13:A>G-13:A>G	598521397	1.94E-09	1	2
	4D	10	109431634|F|0-54:G>A-54:G>A	370691683	4.78E-09	1	3
	6D	11	108582786|F|0-49:T>G-49:T>G	143959936	1.42E-09	1	2
						Total	**20**
DI	2B	1	108980638|F|0-33:T>C-33:T>C	584134131	2.76E-08	1	1
	2D	2	107489027|F|0-34:C>T-34:C>T	302776397	1.41E-07	1	1
	5B	3	109305833|F|0-34:C>G-34:C>G	526249013	4.31E-07	1	1
	7A	4	108206445|F|0-49:C>A-49:C>A	67617920	5.04E-08	2	1
		5	107952026|F|0-63:G>C-63:G>C	498861613	3.70E-07		1
	7D	6	109035950|F|0-29:C>T-29:C>T	88318125	1.89E-07	1	1
							**6**

Table S2. Other genes and proteins identified by GEA with seven climatic variables.

<table>
<tr><th>Chr</th><th>SNP</th><th>Gene</th><th>Variable</th><th>Protein</th><th>Function</th></tr>
<tr><td colspan="6">Morphological responses</td></tr>
<tr><td>2B</td><td>107488994|F|0-17:T>C-17:T>C</td><td>TraesCS2B02G060700</td><td>HITmead</td><td>Fbox (FBX).</td><td>Regulation of multiple developmental processes.</td></tr>
<tr><td>2B</td><td>108980638|F|0-33:T>C-33:T>C</td><td>TraesCS2B02G410500</td><td>DI</td><td>Ovate Family Proteins (OFP).</td><td>Morphological regulation in response to stress signals.</td></tr>
<tr><td>4B</td><td>106772473|F|0-11:T>G-11:T>G</td><td>TraesCS4B02G051500</td><td>PS</td><td>Transcription factor SCREAM2 (SCRM2).</td><td>Constitutive stomatal differentiation in the epidermis.</td></tr>
<tr><td>4B</td><td>108145974|F|0-24:T>C-24:T>C</td><td>TraesCS4B02G064000</td><td>HITmead</td><td>Growth regulating factor 1 (GIF1).</td><td>Regulation of cell expansion in foliar tissues and cotyledons.</td></tr>
<tr><td>4D</td><td>108773963|F|0-37:C>T-37:C>T</td><td>TraesCS4D02G117100</td><td>HITmead</td><td>Fbox (FBX).</td><td>Regulation of multiple developmental processes.</td></tr>
<tr><td>6B</td><td>109177937|F|0-11:A>G-11:A>G</td><td>TraesCS6B02G190600</td><td>AP</td><td>ADP-ribosylation factor GTPase (AGD12).</td><td>Calcium-dependent proteins that affect gravitropism in AT.</td></tr>
<tr><td>6B</td><td>109354013|F|0-58:A>G-58:A>G</td><td>TraesCS6B02G269700</td><td>PS</td><td>Similar to OCL1 homeobox (ROC5).</td><td>Modulates the curling of the leaves.</td></tr>
<tr><td colspan="6">Responses to biotic stress</td></tr>
<tr><td>2A</td><td>108514047|F|0-10:A>C-10:A>C</td><td>TraesCS2A02G499400</td><td>PS</td><td>Pelota homolog (PELO).</td><td>Defense responses to bacteria.</td></tr>
<tr><td>3A</td><td>108028914|F|0-20:A>G-20:A>G</td><td>TraesCS3A02G329100</td><td>MaxT and HITmax</td><td>rRNA N-glycosidase (RIP).</td><td>Antibacterial and antifungal activity.</td></tr>
<tr><td>3B</td><td>108146856|F|0-65:T>A-65:T>A</td><td>TraesCS3B02G523300</td><td>HITmead</td><td>Disease resistance (NBS-LRR).</td><td>Disease resistance genes in plants.</td></tr>
<tr><td>3D</td><td>109610030|F|0-20:C>T-20:C>T</td><td>TraesCS3D02G475300</td><td>HITmax</td><td>Protein LIFEGUARD 4 (LFG4).</td><td>Defense response to pathogens.</td></tr>
<tr><td>4A</td><td>108145069|F|0-52:C>A-52:C>A</td><td>TraesCS4A02G447200</td><td>AP</td><td>Disease resistance (NBS-LRR).</td><td>Disease resistance genes in plants.</td></tr>
<tr><td>5B</td><td>108773279|F|0-51:C>T-51:C>T</td><td>TraesCS5B02G349800</td><td>HITmead</td><td>Flavin-containing monooxygenase (FMO).</td><td>Defense response to pathogens.</td></tr>
<tr><td>5B</td><td>109119258|F|0-68:T>C-68:T>C</td><td>TraesCS5B02G400100</td><td>PS</td><td>Cyclic nucleotide-gated ion channel 2 (CNGC2).</td><td>Defense response to pathogens.</td></tr>
<tr><td colspan="6">Photosynthesis</td></tr>
<tr><td>2A</td><td>108020469|F|0-43:T>C-43:T>C</td><td>TraesCS2A02G467700</td><td>PS</td><td>(S)-2-hydroxy-acid oxidase (GLO1).</td><td>Photorespiration and regulation of photosynthesis.</td></tr>
<tr><td>2D</td><td>108968402|F|0-23:C>T-23:C>T</td><td>TraesCS2D02G136200</td><td>AMT</td><td>Magnesium-chelatase subunit, chloroplastic (CHLH).</td><td>Chlorophyll biosynthesis.</td></tr>
<tr><td>4D</td><td>109363420|F|0-34:G>A-34:G>A</td><td>TraesCS4D02G086900</td><td>HITmead</td><td>Rieske (2Fe-2S) domain (PSB33).</td><td>Assembly and stabilization of the PSII.</td></tr>
<tr><td>5D</td><td>107001591|F|0-37:T>C-37:T>C</td><td>TraesCS5A02G437100</td><td>PS</td><td>Photosystem II reaction center L (psbL).</td><td>The PSII core proteins.</td></tr>
<tr><td>7A</td><td>108830300|F|0-9:T>G-9:T>G</td><td>TraesCS7A02G107200</td><td>MaxT</td><td>Gamma carbonic anhydrase 1 (γCA1).</td><td>Photorespiration.</td></tr>
<tr><td>7D</td><td>109035950|F|0-29:C>T-29:C>T</td><td>TraesCS7D02G138100</td><td>DI</td><td>Cytochrome b6-f complex subunit 5 (PETG).</td><td>Electron transfer between PSII and PSI.</td></tr>
</table>

Table S2. Continued.

Chr	SNP	Gene	Variable	Protein	Function
			**Flowering**		
1A	108891114|F|0-33:A>G-33:A>G	TraesCS1A02G220300	AMT, MaxT, HITmead and HITmax.	CONSTANS-like (*COL*).	Regulation of floral development.
2A	109058266|F|0-7:C>T-7:C>T	TraesCS2A02G390500	AP	Casein kinase II subunit beta (*CK2*).	Circadian Regulation.
2B	107488994|F|0-17:T>C-17:T>C	TraesCS2B02G059500	HITmead	Flowering promoting factor 1 (*FPF1*).	Control of flowering time.
3B	108953425|F|0-21:A>G-21:A>G	TraesCS3B02G081800	PS	Adenylyl-sulfate kinase (*APK*).	Circadian Regulation.
			**Nutrient assimilation**		
2A	109058266|F|0-7:C>T-7:C>T	TraesCS2A02G390800	AP	Metal-nicotianamine transporter (*YSL1*).	Iron uptake and transport.
3A	108028914|F|0-20:A>G-20:A>G	TraesCS3A02G329700	MaxT and HITmax	Purple acid phosphatase (*PAP*).	Nutritional deficiencies.
7B	107878167|F|0-16:G>A-16:G>A	TraesCS7B02G093800	MaxT	Starch synthase, chloroplastic/amyloplastic (*wSs2a-3*).	Starch biosynthesis.
